# Recombinant adenovirus as a model to evaluate the efficiency of free chlorine disinfection in filtered water samples

**DOI:** 10.1186/s12985-015-0259-7

**Published:** 2015-02-22

**Authors:** Mariana A Nascimento, Maria E Magri, Camila D Schissi, Célia RM Barardi

**Affiliations:** Laboratório de Virologia Aplicada, Departamento de Microbiologia, Imunologia e Parasitologia, Universidade Federal de Santa Catarina, 88040-900 Florianópolis, Santa Catarina Brazil

**Keywords:** Recombinant GFP-adenovirus, Chlorine, Filtered water, Fluorescence microscopy, qPCR

## Abstract

**Background:**

In Brazil, ordinance no. 2,914/2011 of the Ministry of Health requires the absence of total coliforms and *Escherichia coli (E. coli)* in treated water. However it is essential that water treatment is effective against all pathogens. Disinfection in Water Treatment Plants (WTP) is commonly performed with chlorine.

**Methods:**

The recombinant adenovirus (rAdV), which expresses green fluorescent protein (GFP) when cultivated in HEK 293A cells, was chosen as a model to evaluate the efficiency of chlorine for human adenovirus (HAdV) inactivation in filtered water samples from two WTPs: Lagoa do Peri (pH 6.9) and Morro dos Quadros (pH 6.5). Buffered demand free (BDF) water (pH 6.9 and 8.0) was used as control. The samples were previously submitted to physicochemical characterization, and bacteriological analysis. Two free chlorine concentrations and two temperatures were assayed for all samples (0.2 mg/L, 0.5 mg/L, and 15°C, and 20°C). Fluorescence microscopy (FM) was used to check viral infectivity *in vitro* and qPCR as a molecular method to determine viral genome copies. Real treated water samples from the WTP (at the output of WTP and the distribution network) were also evaluated for total coliforms, *E. coli* and HAdV.

**Results:**

The time required to inactivate 4log_10_ of rAdV was less than 1 min, when analyzed by FM, except for BDF pH 8.0 (up to 2.5 min for 4log_10_). The pH had a significant influence on the efficiency of disinfection. The qPCR assay was not able to provide information regarding rAdV inactivation. The data were modeled (Chick-Watson), and the observed Ct values were comparable with the values reported in the literature and smaller than the values recommended by the EPA. In the treated water samples, HAdV was detected in the distribution network of the WTP Morro dos Quadros (2.75 × 10^3^ PFU/L).

**Conclusion:**

The Chick-Watson model proved to have adjusted well to the experimental conditions used, and it was possible to prove that the adenoviruses were rapidly inactivated in the surface water treated with chlorine and that the recombinant adenovirus expressing GFP is a good model for this evaluation.

## Background

Currently, enteric viruses are considered to be the main etiological agents of waterborne diseases, accounting for 30-90% of gastroenteritis worldwide [1]. Enteric viruses are frequently aggregated in the environment [2], and due to the small size of the particles (0.5 – 1.0 μm), they are not efficiently retained in the filtration stage at Water Treatment Plants (WTPs) [3]. Disinfection is therefore critical for reducing the infectious virus concentrations in source water.

According to the guidance manual published in 1991 by the Environmental Protection Agency of the United States (US EPA), a 4log_10_ (99.99%) removal or inactivation of enteric viruses by filtration and/or disinfection is recommended. The EPA also recommends values for the contact time - Ct (disinfectant concentration (mg/L) x time (min)) of 4, 6 and 8 to achieve inactivation of 2log_10_, 3log_10_ and 4log_10_, respectively, using free chlorine [4]. However, the values established in this manual were based on studies with hepatitis A in buffered demand free water at 5°C. As the water quality can significantly affect the effectiveness of the disinfection by free chlorine [5], it is unclear whether these recommended Ct values are sufficient to inactivate other viral pathogens in different water matrices.

Enteric viruses are generally more resistant to environmental conditions and conventional water treatment using chlorination and filtration than enteropathogenic bacteria, and there is no potential for replication in the environment because the viruses are obligatory intracellular parasites. Although virus degradation is expected to occur, the amount of virus that remains is more meaningful than the amount of remaining bacteria that can re-grow after being excreted. There have been virus-related outbreaks with the consumption of water in compliance with bacterial standards [6].

The human adenovirus (HAdV) belongs to the Adenoviridae family, genus *Mastadenovirus*, comprising 57 serotypes [7]. HAdV has been indicated as a potential marker of human fecal contamination in water [6]. The current contaminant candidate list of the aquatic environment (CCL3) considers the adenovirus as a high priority emerging contaminant present in drinking water and a candidate contamination marker of the aquatic environment [8].

HAdV has been extensively detected in environmental matrices. In 2005, Choi and Jiang [9] found that 16% of the river samples in California, USA were positive for HAdV (10^2^ - 10^4^ gc/L). Albinana-Gimenez et al. in 2009 [10] described that 90% of the river water samples in Barcelona, Spain were HAdV positive (10^1^ - 10^4^ gc/L). Dong et al. in 2010 [11] detected HAdV in 100% of the sewage samples (1.87 × 10^3^ - 4.6 × 10^6^ gc/L) and in 83.33% of the recreational water samples (1.7 × 10^1^ – 1.19 × 10^3^ gc/L) in New Zealand. Win-Jones et al., in 2011 [12] found that 60.6% of the European recreational and fresh water samples were positive for HAdV, with a mean value of 3.260 × 10^3^ gc/L. In 2012, Fongaro et al. [13] described an HAdV presence in 96% of the samples collected in the Peri Lagoon, Brazil (1.73 × 10^6^ - 2.41 × 10^8^ gc/L) and Garcia et al. (2012) [14] described a presence of HAdV in 100% of the river water samples in Brazil, with an average of 10^7^ gc/L. In the same year, Ye et al. [15] described 100% HAdV positive for river and drinking water samples in Wuhan, China (10^2^ - 10^4^ gc/L).

Several studies have evaluated the inactivation efficiency of HAdV by free chlorine in buffer [3,16,17] in waters from rivers and lakes [5], groundwater [3], seawater [18] and sewage [19]. However, the methods chosen to evaluate the HAdV infectivity are often time-consuming. The plaque assay has long been considered a standard method, although it can require 5 to 12 days to achieve results [5,17-19]. Other methods are based on genome detection, such as PCR or the observation of a cytopathic effect.

As an alternative, recombinant adenoviruses (rAdV) can be used as a viral model to study the water disinfection procedures. rAdV are defective in their replication, as they lack the early gene, E1, which is involved in viral gene transcription, DNA replication, and the inhibition of host cell apoptosis [20]. Thus, rAdV replication is weakened in this condition, unless the replication occurs in permissive cell lines that express the E1 gene products, such as the Human Embryonic Kidney (HEK) 293A cells [21]. rAdV replication can, therefore, be directly monitored by fluorescence methods, based on the expression of the green fluorescent protein (GFP) that is encoded by a gene incorporated into the viral DNA. HEK 293A cells infected with rAdV provide a novel reporter for viral infectivity assays, enabling the use of rapid (24 h) and quantitative methods of monitoring GFP expression in individual cells, such as fluorescence microscopy.

In this context, the goal of the present study was to evaluate the viral inactivation in water collected from two Water Treatment Plants after the filtration (non-disinfected) by subsequent free chlorine addition, using the recombinant adenovirus as a model. Buffered demand free (BDF) water was used as the control. This study also evaluates the treated water quality throughout the water distribution network in relation to the concentration of human adenovirus and total coliforms.

## Results

### Water quality of the Lagoa do Peri (LP) and Morro dos Quadros (MQ) water treatment plants

The physicochemical parameters of the source waters used in the disinfection experiments are shown in Table 1.

**Table 1:** **Physicochemical parameters of the water samples**

	**LP** ^**a**^	**MQ** ^**b**^
pH	6.9	6.5
Turbidity	1.52 uT	0.7 uT
Temperature	25.3°C	19.1°C
Conductance	53 μS/cm	24.1 μS/cm
Nitrite (NO_2_^−^)	0.56 μg/L	3.98 μg/L
Nitrate (NO_3_^−^)	4.34 μg/L	33.32 μg/L
Ammonia (NH_3_^+^)	16.65 μg/L	34.40 μg/L
Total coliforms	>8.0 MPN/100 mL	4.6 MPN/100 mL
*E. coli*	>8.0 MPN/100 mL	<1.1 MPN/100 mL

^a^LP: Lagoa do Peri Water Treatment Plant.

^b^MQ: Morro dos Quadros Water Treatment Plant.

Samples harvested at the Lagoa do Peri (LP) and Morro dos Quadros (MQ) water Treatment Plants.

### Disinfection assays

To determine the influence of the seeded virus stocks on the chlorine demand, the free chlorine decay was analyzed in the water samples with and without the seeding purified virus stock. No significant differences were found between the two conditions (P > 0.05) (not shown). In general, the concentrations remained constant during the analysis period, showing no significant decay (P > 0.05). The rates of the free chlorine decay, although very low, were used to model the exponential regression by the Chick-Watson model.

No significant log reduction of the viral stock was observed over time for all of the analyzed matrices (P > 0.05) for the positive control (not shown). Thus, any log reduction observed in the experiments using the free chlorine was due to the germicidal efficacy of this compound and not to the exposure of the virus to the experimental conditions (e.g., time, matrix composition, and temperature).

**Figure 1:**
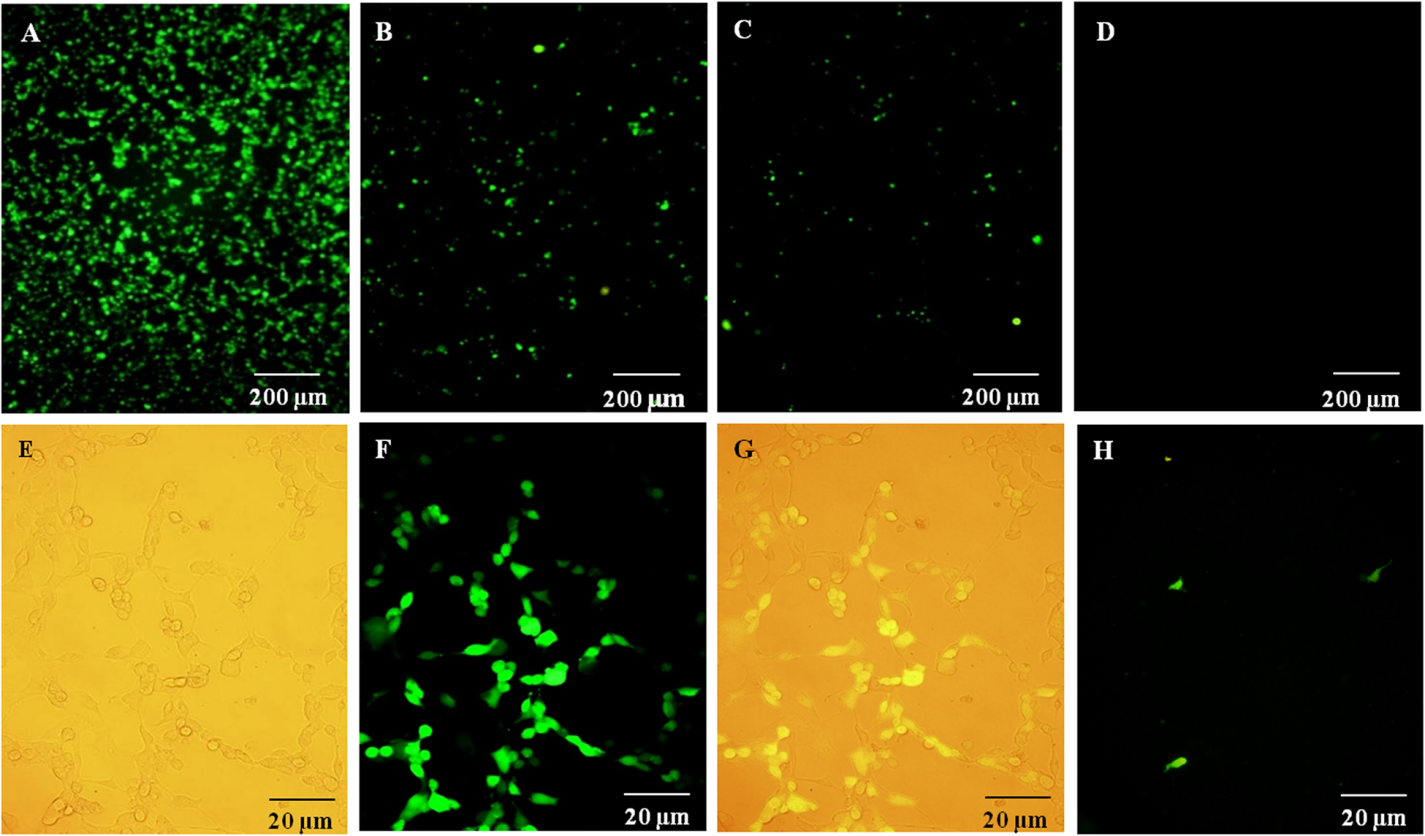
**HEK 293A cells infected with rAdV by fluorescence microscopy and light microscopy.** Viral concentration of 8.3 × 10^5^ FFU/mL **(A)**, 8.3 × 10^4^ FFU/mL **(B)**, 8.3 × 10^3^ FFU/mL **(C)**, and cell control **(D)** by fluorescence microscopy, 40x magnification. Cells infected with 8.2 × 10^5^ FFU/mL by light microscopy **(E)**, fluorescence microscopy **(F)** and merged **(G)**, and an example of 3 green fluorescent cells considered to determine the viral titer **(H)**, 400x magnification.

The titer of the purified virus stock was 9 × 10^8^ FFU/mL, enough to observe a 4log_10_ reduction using fluorescence microscopy because the detection limit for this technique was 8.5 × 10^1^ FFU/mL (the virus stock used to determine the detection limit had a titer of 8.5 × 10^7^ FFU/mL). Using this virus stock, the lowest ten-fold dilution that enabled us to count infected fluorescent cells was the 10^−6^ dilution and, for this reason, this dilution was considered the detection limit for this viral titer and for this amount of inoculum (8.5/0.1 mL = 8.5 × 10^1^ FFU/mL). Figure 1 shows the fluorescent pattern of the HEK 293A cells infected with rAdV under fluorescence microscopy.

**Figure 2:**
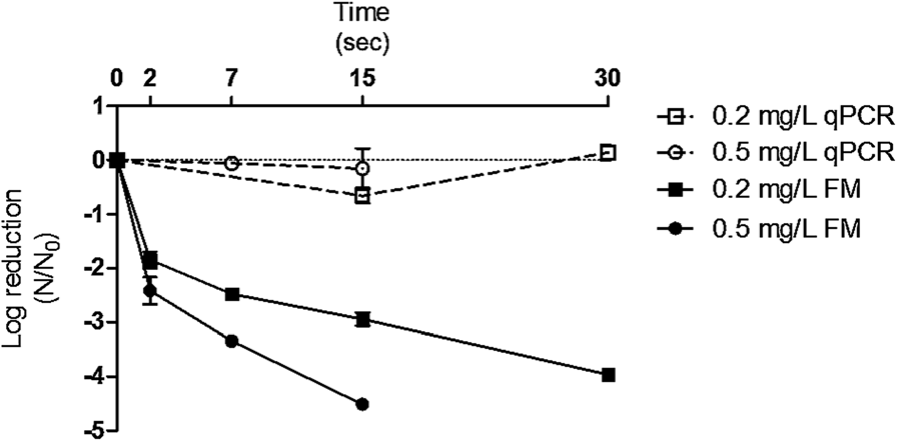
**Inactivation curves of rAdV in BDF buffer pH 6.9.** Temperature of 20°C, free chlorine concentration of 0.2 mg/L and 0.5 mg/L by qPCR and fluorescence microscopy (FM) assays.

**Figure 3:**
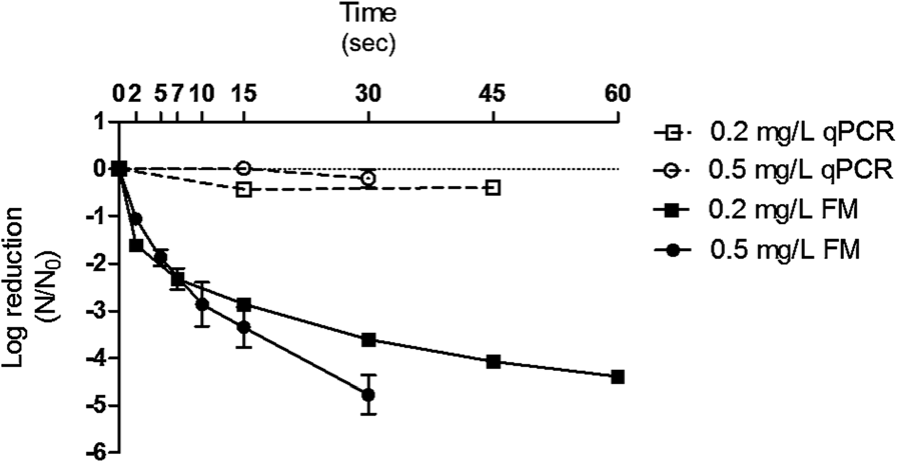
**Inactivation curves of rAdV in Lagoa do Peri water treatment plant.** Sample pH 6.9, temperature of 20°C, free chlorine concentration of 0.2 mg/L and 0.5 mg/L by qPCR and fluorescence microscopy (FM) assays.

**Figure 4:**
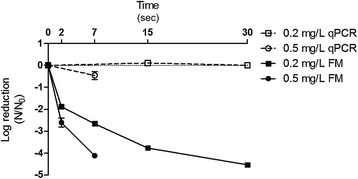
**Inactivation curves of rAdV in Morro dos Quadros water treatment plant.** Sample pH 6.5, temperature of 20°C, free chlorine concentration of 0.2 mg/L and 0.5 mg/L by qPCR and fluorescence microscopy (FM) assays.

**Figure 5:**
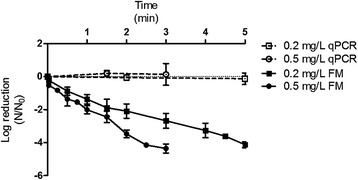
**Inactivation curves of rAdV in BDF buffer pH 8.0.** Temperature of 20°C, free chlorine concentration of 0.2 mg/L and 0.5 mg/L by qPCR and fluorescence microscopy (FM) assays.

The free chlorine rAdV disinfection was performed in duplicate, with 0.2 mg/L and 0.5 mg/L free chlorine in the LP and MQ samples and in the BDF buffer pH 6.9 and 8.0, at 15°C and 20°C. A four-log inactivation was attempted for all of the experiments. The time required to inactivate 4log_10_ rAdV was less than 1 min for both concentrations (0.2 mg/L and 0.5 mg/L) of free chlorine when analyzed by fluorescence microscopy (Figures 2, 3 and 4), with the exception of the BDF buffer at pH 8.0, which showed the slowest decay of approximately 2.5 min with 0.5 mg/L of free chlorine to decay 4log_10_ and 5 min with 0.2 mg/L of free chlorine to achieve the same decay (Figure 5).

The MQ water sample (pH 6.5) showed the highest disinfection rate, requiring 7 s to reduce 4log_10_ with 0.5 mg/L and approximately 20 s with 0.2 mg/L at 20°C (Figure 4). In general, the viral inactivation was higher in MQ (pH 6.5) (Figure 4), followed by LP (pH 6.9), the BDF buffer pH 6.9 (Figures 3 and 2) and finally the BDF buffer pH 8.0 (Figure 5). When the influence of temperature was analyzed, no significant difference was found between the experiments performed at 20°C and 15°C (P > 0.05); therefore, only the results obtained at 20°C were expressed graphically. No significant variation of the logarithmic reduction was observed for all experimental conditions when analyzed by qPCR (P > 0.05) (Figures 2, 3, 4 and 5).

No significant difference was found between LP and MQ when compared with the BDF buffer at pH 6.9 (P > 0.05). The experiments conducted with 10 mL and 40 mL also showed no significant difference (P > 0.05).

### Kinetic modeling

The Chick-Watson (CW) model was used to predict the free-chlorine inactivation kinetics of rAdV for each experimental condition. Table 2 lists the parameters estimated by the CW model analysis: *k’* (rate of free chlorine decay), *k* (rate of viral inactivation) and R^2^ (Ln (N/N_0_) observed x Ln (N/N_0_) of the Chick-Watson model).

**Table 2:** **Parameters estimated by Chick-Watson model analysis**

**Water sample** ^**a**^	**Free chlorine (mg/L)**	**k’ (min** ^**−1**^ **)**	**k (min** ^**−1**^ **)**	**R** ^**2**^
BDF pH 8.0 20°C	0.2	0.0001	3.6645	0.9709
	0.5	0.0001	6.1967	0.9719
BDF pH 6.9 20°C	0.2	0.0001	35.9078	0.7348
	0.5	0.0001	82.9889	0.8125
LP 20°C	0.2	0.105	52.0292	0.7852
	0.5	0.397	47.5822	0.9106
LP 15°C	0.2	0.105	72.7319	0.8382
	0.5	0.397	44.3554	0.7655
MQ 20°C	0.2	0.147	100.5580	0.7913
	0.5	0.0001	162.8456	0.8712
MQ 15°C	0.2	0.147	88.9608	0.7844
	0.5	0.0001	75.0303	0.7695

^a^BDF: Buffered demand free; LP: Lagoa do Peri Water Treatment Plant; MQ: Morro dos Quadros Water Treatment Plant.

Values of *k ’*(rate of decay of free chlorine), *k* (rate of viral inactivation) and R^2^ (Ln (N/N0) observed x Ln (N/N0) of Chick-Watson model determined for each experimental condition.

The Ct values (mg/L x min) predicted for the viral inactivation are shown in Table 3. As seen in Figures 2, 3, 4 and 5, the viral inactivation followed the same pattern, with lower Ct values of the 4log_10_ inactivation for MQ (0.067 and 0.101), followed by LP (0.14), the BDF buffer pH 6.9 (0.187) and finally the BDF buffer pH 8.0 (1.87 Ct value; Table 3).

**Table 3:** **Ct values for rAdV inactivation by free chlorine determined by Chick-Watson model**

**Water sample** ^**a**^	**- Log**_**10**_ **inactivation**	***Ct*** **(mg/L x min) model (mean/standard deviation)**	**EPA guidance manual Ct value (1991)**
BDF pH 8.0 20°C	2	0.87/0.17	1
	3	1.49/0.70	2
	4	1.87/0.88	3
BDF pH 6.9 20°C	2	0.016/NO^b^	1
	3	0.091/0.047	2
	4	0.187/0.08	3
LP 20°C	2	0.022/0.005	1
	3	0.06/0.028	2
	4	0.14/0.028	3
LP 15°C	2	0.014/0.005	2
	3	0.06/0.028	3
	4	0.14/0.028	4
MQ 20°C	2	0.005/NO^b^	1
	3	0.027/0.015	2
	4	0.067/0.013	3
MQ 15°C	2	0.017/0.001	2
	3	0.048/0.013	3
	4	0.101/0.033	4

^a^BDF: Buffered demand free; LP: Lagoa do Peri Water Treatment Plant; MQ: Morro dos Quadros Water Treatment Plant.

^b^NO: not observed.

Ct values (mean/standard deviation) calculated for each experimental condition and compared with EPA Guidance Manual.

### Treated water quality

The t-MQ, t-LP, n-MQ and n-LP water samples were analyzed for the total coliforms and *Escherichia coli* (*E. coli)*, the free chlorine concentration (mg/L), and the HAdV viability. The average recovery of the organic flocculation for the virus concentration was 6.4%. The range of the free chlorine concentration was from 0.57 to 4.0 mg/L and is within the standard required by the MH Ordinance 2.914/2011, which defines a minimum of 0.2 mg/L and a maximum of 5.0 mg/L [22]. None of the samples were positive for both the total coliforms and *E. coli*, with values lower than 1.1 MPN/100 mL (sensitivity limit). Among the tested samples, t-MQ showed contamination with 2.75 × 10^3^ PFU/L infectious HAdV (value corrected by recovery), and at this point, the measured free chlorine concentration was 0.57 mg/L. None of the other samples were positive for HAdV, with values lower than 1 × 10^3^ PFU/L (sensitivity limit).

## Discussion

The application of recombinant adenovirus provides a versatile system for therapeutic applications and gene expression studies, including gene transfer *in vitro*, gene therapy and vaccine therapy [20]. Despite this well-established use, we describe herein a novel application of rAdV in the environmental virology field, especially in disinfection assessment studies. The current study shows, for the first time, the efficiency of free chlorine disinfection of rAdV in water samples undergoing treatment for human consumption. The temperatures of 15°C and 20°C mimic the temperature range of the natural waters in south Brazil during the winter and summer seasons [23], respectively, and the pH conditions, which were not modified. We believe that it is very important to select temperatures that assess the real conditions that occur in the real environment. Comparison techniques based on genome detection (qPCR) and infectivity (cell culture) are also important because risk assessment studies based on genomic copy detection are encouraged [24-28], and other studies have reported that free chlorine can damage genetic material [29,30].

The use of rAdV proved to be economical, convenient and fast for several reasons: it does not require the use of primary and secondary antibodies; it decreases the possibility of overestimating the viral titers due to non-specific binding; it avoids cell loss during the washing stages commonly performed in immunodetection techniques; and it is faster (24 h) than the conventional plaque assay method (7 to 10 days), described by Cromeans et al. (2008) [31].

Regarding the GFP fluorescence stability in chlorine solutions, according to Mazzola et al. (2006) [32], who evaluated the GFP stability in chlorinated water and buffered solutions, the main conclusion was that GFP is a suitable fluorescent marker for monitoring disinfection effectiveness. They observed that, with constantly stirred solutions, the GFP fluorescence decreased abruptly after contact with chlorine in concentrations greater than 150 ppm, and the GFP fluorescence intensity was reduced by 42% in the initial 30 s of contact with a 70 ppm phosphate buffered chlorinated solution. Webb et al. (2001) [33] exposed *Aureobasidium pullulans* cells expressing GFP to chlorinated solutions (25–150 ppm) and observed that the loss of GFP fluorescence was highly correlated with a decrease of the number of viable cells. Casey and Nguyen (1995) [34] exposed *Escherichia coli* cells also expressing GFP and observed the same result as Webb et al. (2001). In the present study, the chlorine concentrations employed were 0.2 ppm and 0.5 ppm, much lower than the values described above. Therefore, we can conclude that the GFP fluorescence itself was not affected by this low concentration of applied chlorine, and the lack of fluorescence is certainly due to a lack of rAdV replication. This phenomenon was also proven by the same effect of the chlorine on viral disinfection using non-recombinant human adenovirus, which was previously described in the literature [3,5,17].

Viral purification is essential for the experiments of disinfection by free chlorine because viral suspensions contain considerable amounts of organic matter that consumes free chlorine, preventing its virucidal and bactericidal action [18]. This work was the first to use chromatography as a method of purification and proved to be comparable to studies using other forms of purification, with comparable and adequate Ct values [5,17] because the concentrations of disinfectant did not vary significantly in the presence of the purified virus stock (P > 0.05).

No significant difference in the disinfection efficiency was observed (P > 0.05) between the tested temperatures (15°C and 20°C). However, the pH variation exerted a great influence on the disinfection efficiency: the Ct for the 4log_10_ disinfection at BDF pH 8.0 (1.87) was 10 times greater than the Ct at BDF pH 6.9 (0.187). This result is due to residual free chlorine in both pHs; at pH 8.0 there is approximately 25% HOCl and 75% of the hypochlorite ion (HCl^+^), and at pH 6.9, approximately 80% is HOCl, and 20% is HCl^+^. According to AWWA (2006) [35], the germicidal efficiency of HOCl is approximately 100 times greater than HCl^+^, which explains the observed results; therefore, the pH of the water can cause a variability in the disinfection efficiency.

Nevertheless, fresh water submitted to water treatment is constantly influenced by geological features. It is well known that the levels of chemicals in soils reflect the levels at the source rock, except in cases with anthropogenic influence [36,37], and the geology has a great influence on the chemical characteristics of the soil and surface water [38]. Moreover, it has been demonstrated that the pH values can vary in different water bodies, such as rivers, water reservoirs or estuaries, depending on the season [39-41] and the daily basis [40,42] and spatially (variation throughout the water layer or sampling sites) [42,43]. Furthermore, air pollutants, such as carbon dioxide (CO_2_), have a great influence on the pH of water because air pollutants can enter the water through biological metabolism involving organic carbon and through equilibrium with the atmosphere. Once in the water, CO_2_ reacts and forms bicarbonate (HCO_3_^−^) and carbonate (CO_3_^2−^), decreasing the pH. Therefore, more air pollution results in more CO_2_ in the water and higher water acidity [44]. Some other factors can affect the disinfection efficiency, such as antioxidants from commercial hygiene products, which reduce hypochlorite to chloride ions and decrease the free chlorine available for disinfection [45,46]. In addition, it is well known that the temperature range can influence the pH, as well as the CO_2_ solubility and may vary on a daily basis [47]. Altogether, these factors can affect the pH and change the disinfection dynamics. Thus, it is essential to carefully control the pH in water treatment plants throughout the process, especially before the addition of chlorine, due to its great influence on the disinfection performance.

It is possible to observe that the inactivation curves for all of the experimental conditions, except for the BDF pH 8.0, were characterized by two phases: an initial phase in which the inactivation occurred rapidly (approximately 2log_10_ in 2 seconds), followed by a phase with a lower rate of inactivation, which may be designated the “tailing phase.” Page et al. (2009) [16] described that the loss of disinfection efficiency observed during the tailing phase is most likely due to the rapid change of specific chemical moieties on the viral structure that preferentially react with HOCl. Thus, some authors propose that the HOCl-mediated transformation of proteins, which is due to the high reactivity with proteins and their abundance in biological systems, plays a key role in the loss of the biological function of this form of the free residual chlorine, leading to the formation of the tailing phase [48]. As the main feature, adenovirus capsids are composed of proteins (fibers, pentons and hexons) that are physically exposed to the disinfectant. These proteins contain functional groups, such as amines and thiols, that react with free chlorine, leading to a loss of the biological function of the disinfectant [49].

The inactivation curve in the BDF pH 8.0 experiments may be associated with damage involving secondary oxidizing agents [16]. In addition, at this pH, the HOCl concentration is approximately 25% [35], making the disinfection slower with no biphasic behavior observed.

The qPCR assay has already proven to be fast and specific for the detection and quantification of rAdV genomes. However, this technique does not provide sufficient information about inactivated viruses compared with the fluorescence microscopy technique after cell culture. The time necessary for the assays was determined by the disinfection achievement. Therefore, once the 4log_10_ of disinfection was achieved, the experiment was considered concluded, although the viral genomic copies did not show a significant log reduction. In fact, some studies had performed viral disinfection studies employing PCR, and some studies indeed showed a reduction of the viral copies. Nevertheless, they observed the same profile: the genome integrity decreased more slowly than the viral viability [18,19,30,50-54]. Although some studies have reported that free chlorine can damage the viral genetic material [30] by interacting with the amine group of nucleotides [29], it is suggested that the extent of DNA damage caused by free chlorine is not sufficient to detect viral inactivation by the qPCR technique, which often results in very small amplicons [55]. Even with qualitative PCR using primer sets that generate greater amplicons (400 bp to 1,215 bp), the genome integrity is not correlated with the viability of HAdV because the PCR products are generated even when higher concentrations of chlorine are used [30]. This result suggests that the ability of free chlorine to cause damage in the viral genome is limited [30], and viruses with lesions in the capsid proteins caused by chlorine may still contain their genomes that are protected from the inactivation procedures. Thus, the viral nucleic acids detected by PCR or qPCR can be derived from infective and non-infective damaged viruses and from free nucleic acids from lysed viruses, and the results obtained by cell culture, when possible, are more representative of the actual health risk. Therefore, risk assessment studies based on genomic copy detection are inadequate, overestimating the actual risk of consuming drinking water treated with chlorine.

The absence of viable HAdV in the output of both WTPs and the presence of infectious virus in the distribution network suggest that the water treatment is efficient for the inactivation of HAdV; however, the water is re-contaminated during its distribution and/or storage. Thus, low concentrations of residual free chlorine throughout the water distribution system are not sufficient to inactivate high viral loads that are accidentally re-introduced after the water treatment process [3].

It is postulated that biofilms in the drinking water distribution networks may play a role in the accumulation, protection and dissemination of pathogens [56]. The formation of biofilms has been described to provide bacteria much greater resistance to free chlorine, and it has also been shown that viruses can adsorb into biofilms [57]. The detection of viable HAdV in the distribution network may be due to viral aggregation and adsorption by particles, which have previously been reported as increasing the resistance to chlorine and the environment [2,3] or adsorption into biofilms, also protecting them from the action of the free chlorine disinfectant.

As described in the literature, the human adenovirus is rapidly inactivated by free chlorine. However, it is difficult to make a direct comparison of the Ct values due to variations in the experimental conditions, especially the technique used for the viral purification [5]. Thurston-Enriquez et al. (2003) [3], Kahler et al. (2010) [5], and Cromeans et al. (2010) [17] reported Ct values similar to those observed in the present study, except for BDF at pH 8.0, which was reported previously as 0.24 to inactivate 4log_10_ [3], in contrast to the 1.87 value observed in this study. However, the Ct value of 0.24 that was described by Thurston-Enriquez et al. (2003) [3] was calculated by the model, whereas the Ct observed in the experiment (not modeled) was 36.09 for the 4log_10_ reduction. Taken together, these data indicate the high susceptibility of the human adenovirus to free chlorine. Because the present work employed rAdV, and the Ct values reported in the literature for HAdV are comparable, this result confirms the applicability of rAdV as a model for HAdV for studies of free chlorine disinfection and discards the need to perform all experiments with HAdV in parallel in this study. By comparing the Ct values recommended by the EPA [4] with those predicted by modeling, the values are lower than those recommended in all of the experimental conditions, providing a margin of safety for free chlorine water treatment in terms of the human adenovirus.

The Chick-Watson model was chosen as it best fits the reactors in the batch mode or ideal piston, in which the longitudinal dispersion is equal to zero [58]. As the experiments were performed in 10 or 40 mL, the longitudinal dispersion is disregarded. The values of *k* (inactivation constant rate) calculated by the Chick-Watson model are in agreement with what was observed: the inactivation was faster in MQ, followed by LP/BDF pH 6.9 and finally BDF pH 8.0 (Table 2 and Figures 2, 3, 4 and 5) because the *k* value is directly proportional to the inactivation rate.

The constants of the Chick-Watson model described herein (*k, k’*) were determined by conducting bench experiments. These constants are considered characteristic for the rAdV inactivation kinetics, the specific conditions of the pH and temperature, and the composition of the environmental matrices. Therefore, these constants can be used to calculate the Ct at other chlorine concentrations, without the need to perform additional bench experiments.

## Conclusion

The concentrations of chlorine (0.2 mg/L and 0.5 mg/L) applied to the filtered surface water were effective in inactivating the recombinant adenovirus, which proved to be highly susceptible to chlorine under the conditions studied. The factor that most influenced the disinfection was the pH. The rAdV proved to be a suitable model for this assessment, comparable to the results described in the literature in relation to the non-recombinant human adenovirus. Additionally, the matrix composition did not seem to interfere with the disinfection efficiency. The detection of viable HAdV in the supply network suggests a re-contamination of the water, and the residual chlorine concentration may not be sufficient to inactivate the viruses that have been introduced after the water treatment process.

## Materials and methods

### Virus and cell line

The recombinant human adenovirus (rAdV) serotype 5 was propagated in HEK 293A cells, which were maintained in growth medium composed of Dulbecco’s Modified Eagle Medium (DMEM 1X), supplemented with 10% of fetal bovine serum (FBS) and 1% of HEPES. Five hundred milliliters of the virus stock was produced by a host cell infection using a multiplicity of infection (MOI) of 5. After 24 h of incubation, the flasks were freeze-thawed three times and centrifuged at 3,500 × g for 15 min. The supernatant was recovered and submitted to viral purification using the Vivapure® AdenoPack Stedin Sartorius™ 500 commercial kit.

Briefly, 500 mL of the infected cell supernatant was treated with 12.5 U.mL^−1^ of Benzonase® for 30 min at 37°C, with the aim of degrading the nucleic acids from the cells and the viral particles. After adding Loading Buffer™, this suspension was pumped at 10 mL/min through the chromatography filter and, based on the specific surface viral characteristics, the virus particles were specifically retained. The Washing Buffer™ was then used to remove the contaminants, and 10 mL of the Elution Buffer™ at 1 mL/min was used to elute the viruses from the filter. The eluate was reconcentrated by *Vivaspin 20* by centrifugation at 6,000 *×* g for 15 min, or until the virus stock could be concentrated at approximately 1 mL. With the addition of 9 mL of phosphate-buffered saline (PBS), the purified virus stock was stored at −80°C in aliquots of 0.1 mL.

The human adenovirus 2 (HAdV2) was propagated in a continuous line of A549 cells (permissive cells derived from human lung carcinoma cells, European Collection of Cell Cultures). These cells were kindly donated by Dr. Rosina Gironès from the University of Barcelona, Spain. The A549 cells were propagated in growth medium, consisting of Dulbecco’s Modified Eagle Medium high glucose (DMEM ↑G 1X), supplemented with 5% of fetal bovine serum (FBS) and 1 mM of sodium pyruvate.

### Treated water quality – collection and concentration

These samples were collected to evaluate the quality of the treated water in relation to the HAdV viability, the total coliforms and the *E. coli*. For this evaluation, 4 L of treated water was collected from the Lagoa do Peri (WTP LP) and Morro dos Quadros (WTP MQ) Water Treatment Plants, henceforth referred to as the “treated LP” (t-LP) and “treated MQ” (t-MQ), respectively. Another 4 L water samples were collected from one point in each of the two distribution network supplied by its respective WTP. The samples from the distribution network were collected from a tap at the Universidade Federal de Santa Catarina that was supplied by the WTP MQ and from a residence supplied by the WTP LP, here designated as “network LP” (n-LP) and “network MQ” (n-MQ). All four of the samples were previously treated with 10% sodium thiosulfate and submitted to an analysis of the total coliforms and *E. coli* and then subjected to the flocculation method for virus concentration, as described by Calgua et al. 2013 [59].

The samples were placed in 2 liter-glass beakers (2 beakers per sample), 1.5 g/L of sea salts (SeaSalts - Sigma) were added, and the pH was adjusted to 3.5 with a 1 N HCl solution. A suspension of HAdV (1 × 10^7^ PFU) was added to one of the beakers to assess the viral recovery. Twenty milliliters of skimmed milk solution at pH 3.5 (Pre-flocculated Skimmed Milk, 0.1% - Difco) prepared in artificial seawater (1.5 g/L SeaSalts - Sigma) was added to each beaker. Over a period of 8 h of stirring, the flocks of the acid milk provide a proper surface for viral adsorption and, after 8 h of resting these flocks settle. The supernatant was aspirated, the precipitate was centrifuged at 7000 × g for 30 min at 4°C, and the pellet was resuspended in 10 mL of phosphate buffer (NaH_2_PO_4_, Na_2_HPO_4_, 0.2 M, 1:2 v/v, pH 7.5). The final concentrate was immediately submitted to the plaque assay.

### Plaque assay

The t-LP, t-MQ, n-LP and n-MQ concentrated samples were treated with 1% PSA and inoculated in triplicate in a non-cytotoxic dilution in A549 cells for the plaque assay, as described by Cromeans et al. 2008 [31], with minor modifications (using 0.6% Bacto-agar). Afterwards, the cells were stained with 20% Gram’s crystal violet, the plaques were counted, and the results were expressed in Plaque Forming Units per liter (PFU/L). The theoretical limit of sensitivity limit of this method is 1 × 10^3^ PFU/L.

### Disinfection assays

#### Tested waters

Water samples undergoing treatment were obtained at the Lagoa do Peri Water Treatment Plant (LP) located at Peri Lagoon, city of Florianópolis and the Morro dos Quadros Water Treatment Plant (MQ), located in Palhoça city. Both cities are located in Southern Brazil in the State of Santa Catarina. The water samples were collected after regular treatment (a filtration step) and immediately prior to chemical disinfection. Ten liters of each sample was collected, aliquoted in 500 mL, and stored at −20°C.

Experiments were also conducted with buffered demand free (BDF) water, prepared by dissolving 0.54 g of Na_2_HPO_4_ (anhydrous) and 0.88 g of KH_2_PO_4_ (anhydrous) per liter of deionized, chlorine demand-free water. The pH was adjusted to 8.0 and 6.9 by adding 1 M KH_2_PO_4_. The BDF water was stored in chlorine-demand-free bottles at 4°C until use.

#### Physicochemical parameters and fecal contamination analysis

Using a multiparameter probe (YSI-85), the LP and MQ samples were submitted to physicochemical analysis *in situ* to determine the temperature, conductivity, and pH. In the laboratory, the samples were analyzed for turbidity, nitrite (NO_2_^−^) [60], nitrate (NO_3_^−^) [61], and ammonia (NH_3_) [62]. The nutrients were measured in the filtered water samples using a Millipore AP40–47 mm glass fiber.

A fecal contamination analysis was performed using a commercial *Aquatest Coli – ONPG MUG Laborclin.* One hundred milliliters of the MQ, LP, t-LP, t-MQ, n-LP and n-MQ samples was analyzed, aliquoted in 5 tubes of 20 mL and incubated for 24 h at 35 ± 2°C. The number of positive tubes was counted, and the results were expressed as the Most Probable Number (MPN) of total coliforms per 100 mL. The sensitivity limit of this technique is 1.1 MPN/100 mL.

#### Reagents and glassware treatments

The glassware was made chlorine demand-free, as previously described [3]. The beakers were soaked overnight in a solution of 100 mg/L free chlorine, rinsed with chlorine demand free water and baked for 2 h at 200°C. Following this initial treatment, only a soaking in free chlorine and rinsing in the demand free water were performed. For all of the disinfection experiments, a 2.5% sodium hypochlorite solution (2.38 g/L of free chlorine) was used, which is suitable for the disinfection and treatment of drinking water, in accordance with the Brazilian regulations (Ministry of Health 3.1862.0001 MS). A chlorine stock solution of 100 mg/L was prepared, and a dilution in the BDF of this stock solution was performed to achieve the initial concentration of free chlorine to be used in the disinfection experiments (0.2 mg/L and 0.5 mg/L).

#### Experimental design for disinfection

The viral inactivation was performed with 0.2 mg/L and 0.5 mg/L of the free chlorine because the minimum concentration required by the MH 2.914/2011 is 0.2 mg/L [22]. The temperatures selected were 15°C and 20°C, for 0.2 mg/L and 0.5 mg/L of the free chlorine, except for the experiments with the BDF buffer, which were only performed at 20°C. The volume used in the experiment was 10 mL to be able to observe a 4log_10_ decay (100 μL viral inoculums - 9 x 10^7^ FFU), although the same experimental conditions were also performed with 40 mL (400 μL viral inoculums – 3.6 x 10^8^ FFU) (Table 4). All of the experiments were performed in duplicate.

**Table 4:** **Experimental design for disinfection**

**Water sample/Control** ^**a**^	**Volume used in the experiment (mL)**	**pH**	**Temperature (°C)**	**Chlorine concentration (mg/L)**
LP	10	6.9	15/20	0.2
				0.5
LP	40	6.9	20	0.2
MQ	10	6.5	15/20	0.2
				0.5
MQ	40	6.5	20	0.2
BDF	10	8.0	20	0.2
				0.5
BDF	10	6.9	20	0.2
				0.5

^a^LP: Lagoa do Peri Water Treatment Plant; MQ: Morro dos Quadros Water Treatment Plant; BDF: Buffered demand free.

Treatment applied to each sample, according to volume, pH, temperature and chlorine concentration.

##### Free chlorine decay in water samples

All of the experiments that evaluated the free chlorine decay were performed with 50 mL, because the technique (DPD method - *HANNA Instruments* (HI 95711)) used requires aliquots of 10 mL per analysis point.

In the glassware chlorine-free beakers, 50 mL of the LP, MQ and BDF buffer (pH 6.9 and 8.0) with 0.2 mg/L and 0.5 mg/L of the free chlorine were stirred at 20°C, and the free chlorine concentration was determined at a minimum at the beginning (time 0 s) and at the end of the disinfection time by the DPD method, using the *HANNA Instruments* (HI 95711).

##### Free chlorine decay in water samples with virus stock

For this assay, the same procedure was performed as described in section 2.4.4.1; however, this time with the addition of 500 μL of the purified virus stock.

##### Positive controls

In the glassware chlorine-free beakers, 10 mL of the LP, MQ and BDF buffer (pH 6.9 and 8.0) with 100 μL of the purified virus stock were stirred at 20°C. Four-hundred microliter aliquots were taken at the selected time points (0 s, 30 min and 60 min), kept on ice until the last time points were taken and analyzed by fluorescence microscopy and qPCR for the presence of viruses and the viability tests.

##### Viral inactivation by free chlorine

In the glassware chlorine-free beakers, 10 mL of the LP, MQ and BDF buffer (pH 6.9 and 8.0) with 100 μL of the purified virus stock with 0.2 mg/L and 0.5 mg/L of the free chlorine were stirred at 15°C and 20°C. Four-hundred microliter aliquots were taken, and the residual free chlorine was immediately quenched by placing the samples into collection tubes containing a sterile 10% sodium thiosulfate solution. The samples were kept on ice until the last time point was taken and analyzed by fluorescence microscopy and qPCR for the presence of viruses and the viability.

The same protocol was repeated using 40 mL of the water matrix and 400 μL of the purified virus to certify that the results obtained using minor water volumes (10 mL) do not exhibit any significant difference when compared with larger volumes, as used by some authors in the literature [3,5,17].

#### Evaluation of viral inactivation by cell culture methods

##### Cytotoxicity tests

The cytotoxicity tests were performed to evaluate the potential toxicity caused by the LP and MQ samples in the HEK 293A cells to be used during the disinfection studies. The HEK 293A cell monolayers (1.87 x 10^5^ cells/well) were propagated in 24-well plates (TPP, Switzerland) for 24 h at 37°C in 5% CO_2_. The growth medium was discarded, and an inoculum of 100 μL/well of the pure or diluted samples (1:2, 1:4, 1:8, or 1:16) was prepared in serum-free culture medium (DMEM 1X, 1% PSA - 10 U/mL penicillin, 10 μg/mL streptomycin, 2 ng/mL amphotericin B) and adsorbed into the cells. After 1 h of incubation at 37°C in 5% CO_2_, 650 μL of the maintenance medium (DMEM 1X, containing 2% FBS, 1% HEPES, and 1% PSA) was added. After 24 h, the cell monolayers were observed under an inverted light microscope. These cells were fixed and stained with 0.1% crystal violet to establish a first non-cytotoxic dilution for use in further viral infectivity assays.

Cytotoxicity tests of the t-LP, t-MQ, n-LP and n-MQ samples were performed in the A549 cell monolayers (2.5 × 10^5^ cells/well), as described for the HEK 293A cells, with the exception of the maintenance medium (DMEM ↑G 1X, 2% FBS and 1% PSA), and the monolayers were monitored for 7 days, until the staining.

##### Fluorescence Microscopy (FM)

The HEK 293A cells were grown to obtain confluent monolayers (1.5 x 10^5^ cells/well) in a 48-well plate for 24 h at 37°C in 5% CO_2_. The growth medium was discarded, and 100 μL of each water dilution with 1% PSA was inoculated in duplicate. The cells were incubated for 1 h, and then 400 μL of the maintenance medium was added. After 24 h p.i., the cells were then observed under an epifluorescence microscope with UV light (Olympus). The viral titer was determined by the following formula: (average green cells counted x reciprocal dilution)/inoculum (mL). The results are shown in Focus Forming Units per milliliter (FFU/mL). Figure 1H displays an example of the infected green fluorescent cells that were counted and considered to determine the viral titer.

#### Evaluation of viral inactivation by molecular methods

##### Nucleic acid isolation and quantitative PCR (qPCR)

The extraction of the viral nucleic acid was performed using a commercial QIAmp MinElute Virus Spin Kit (Qiagen, Valencia, CA, USA), according to the manufacturer’s instructions. The nucleic acid was eluted in 60 μL of the elution buffer and stored at −80°C until use for the real-time PCR (quantitative PCR).

For the detection of rAdV, quantitative PCR was performed, as described by Hernroth et al. 2002 [55]. The reaction contained 1:10 dilutions of each sample and the TaqMan PCR master mix (Applied Biosystems, Foster City, CA), along with the primers and TaqMan probes at a volume of 25 μL. All of the amplifications were performed in the StepOne Plus® Real-Time PCR System (Applied Biosystems). Each sample was analyzed in triplicate. For each plate, four serial dilutions of the standard were run in triplicate for each assay, and the genome copies (gc) were measured. Ultra-pure water was used as the non-template control for each assay.

#### Kinetic modeling and statistical analysis

The statistical analyses were performed using GraphPad Prism version 5.0 (USA). Student’s t test was performed, and all significant differences are quoted for P < 0.05. The data were previously confirmed for a normal distribution fitting.

The chlorine decay constant (*k’*) for each experiment was calculated using Microsoft Excel 2007, according to the following equation:

$$ \mathrm{C}\left(\mathrm{t}\right)={\mathrm{C}}_0\  \exp \left(-k't\right) $$

where C(t) and C_0_ is the concentration of the free residual chlorine (mg/L) at time t and at time 0, respectively, and *k’* is the first-order decay rate constant (min^−1^).

The values of the viral inactivation observed by fluorescence microscopy (FFU/mL) were subjected to the previously described Chick-Watson model to predict the contact time:

$$ \mathrm{L}\mathrm{n}\ N/{N}_0= - \mathrm{k}/\mathrm{k}'\mathrm{n}\ \left({{\mathrm{C}}_0}^{\mathrm{n}}\hbox{--} {{\mathrm{C}}_{\mathrm{t}}}^{\mathrm{n}}\right) $$

where *Ln N/N*_*0*_ is the natural logarithm of the survival rate (concentration of viable virus at time t divided by the concentration at time 0), *k* is the inactivation constant rate and *n* is the coefficient of dilution. The *K*’ value of 0.0001 was adopted when the decay of the disinfectant was considered negligible [3], and the *n* value was considered 1 [63].

The Ct values (mg/L × min) predicted for the viral inactivation were determined by multiplying the time (min) and the concentration of free chlorine (C_t_^n^) at each time interval that were calculated by the Chick-Watson model when an approximate 2log_10_, 3log_10_, and 4log_10_ inactivation occurred.
